# A Three Days’ Western Experience

**Published:** 1881-10

**Authors:** J. N. Navin

**Affiliations:** Veterinary Editor Indiana Farmer


### VI.—A THREE DAYS' WESTERN EXPERIENCE.

Editor of Journal of Comparative Medicine:

On Thursday of last week I answered a telegram to Columbus, this State, where I found a terrible and destructive disease among 300 steers and heifers fed at the starch factory. Seventeen had died prior to my getting there, and a like number were sick. I had many of them caught, and found their pulses ranging from 60 to 70 and 80, according to the time they were affected. I determined to kill one, the one farthest advanced in the disease, a heifer; pulse 108; did not bleed her; found every organ unsound except the lungs; the spleen, on the surface a dark muddy brown, on the inner surface a brown-black, resisting pressure by the finger no more than would soft mush. The kidneys were darker than normal, and when cut into presented a black surface containing bloody water, while the substance was broken down like that of the spleen. The liver, though not so soft, was highly discolored (dark), and the gall-bladder, though not as large as many I have seen, contained bile as black as ink. The bladder contained a dark bloody fluid, such as equal parts of blood and water would present, but vastly darker. The entire line of the bowels showed inflammation, and the peritoneum a greenish yellow. The heart was pale-colored and flabby. Nothing appeared in the bowels except the starch offal. I ordered the starch feed discontinued, and for the sick animals tincture of aconite and of belladonna to reduce the circulation, and podophyllin and calomel to regulate the liver. I also added spirits of nitre as a diuretic, and am waiting the result. Of course very little may be expected from the very sick ones. Could you throw any light upon the cause? Is it malaria or the result of feeding too much corn stuff and no hay or straw?

Friday I answered another dispatch, forty miles from here. A horse ran off with a buggy and broke the upper pastern bone. He rested on the heels of his shoe, the toe elevated. I had the shoe taken off and one put on reaching back four inches from the heel; put on the heels at extreme ends, turned up half an inch. Still his pastern, on pressure, descended down near the heels ot shoe. I had a block of timber fashioned to fit under the heel, with two holes in it to receive the turned-up heels of the shoe, with a pledget of muslin for the pastern to rest on, and left him resting quietly on it; ordered constant cold water and tincture of arnica applications. I had an extraordinary case on Saturday. Will write you again.

J. N. Navin, V.S.,
*Veterinary Editor Indiana Farmer.*

